# ^99^Tc^m^-N(NOEt)_2_在KMB17人胚肺二倍体细胞与不同种类肺癌细胞中的摄取动力学比较

**DOI:** 10.3779/j.issn.1009-3419.2010.04.07

**Published:** 2010-04-20

**Authors:** 铁昆 马, 剑鸣 曹, 伟 贾

**Affiliations:** 1 650032 昆明，昆明医学院第一附属医院核医学科 Department of Nuclear Medicine, the First Affiliated Hospital of Kunming Medical College, Kunming 650032, China; 2 650032 昆明，昆明医学院第一附属医院临床实验中心 Center of Medical Research, the First Affiliated Hospital of Kunming Medical College, Kunming 650032, China

**Keywords:** 肺肿瘤, 肺癌细胞, 人胚肺二倍体细胞KMB17, N(NOEt)_2_, 药代动力学, Lung neoplasms, Lung tumor cell, Human embryonic lung diploid fibroblasts KMB17, N(NOEt)_2_, Pharmacokinetics

## Abstract

**背景与目的:**

PET/CT显像价格昂贵，因此研究适合SPECT/CT的肿瘤显像剂显得尤为重要。^99^Tc^m^N(NOEt)_2_[^99^Tc^m^-氮-二（N-乙基-N-乙氧基二硫代氨基甲酸盐）]可被肺肿瘤细胞等摄取。本文旨在细胞水平对比研究其在KMB17人胚肺二倍体成纤维细胞与多种肺癌细胞中的摄取动力学差异，探讨^99^Tc^m^-N(NOEt)_2_显像对肺肿瘤的鉴别诊断价值。

**方法:**

在等体积且细胞浓度为1×10^6^/mL的YTMLC个旧人肺鳞癌细胞、SPC-A1人肺腺癌细胞、AGZY低转移人肺腺癌细胞、973人高转移肺腺癌细胞、GLC-82个旧人肺腺癌细胞以及KMB17人胚肺二倍体成纤维细胞的混悬培养液中分别加入等量^99^Tc^m^-N(NOEt)_2_，按300 μL精确取样分装于试管中，37 ℃恒温培养，均分别于5 min、15 min、30 min、45 min、60 min、75 min、90 min离心沉淀，测定细胞内放射性计数，计算各时间点各种细胞对^99^Tc^m^-N(NOEt)_2_的摄取百分率。

**结果:**

6种细胞除SPC-A1细胞与973细胞之间的差异无统计学意义外（*LSD-t*检验，*P*=0.838），其余两两之间的差异均有统计学意义（*P* < 0.001）；细胞的摄取率大小关系为：973与SPC-A1 > YTMLC > GLC-8- > AGZY > KMB17；30 min-45 min时摄取率逐渐趋于平稳，45 min摄取率均大于其摄取峰值的96.6%。

**结论:**

^99^Tc^m^N(NOEt)_2_显像在肺肿瘤的鉴别诊断中有一定的临床应用价值，且早期显像在30 min左右是适合的。

## Abstract (en)

**Background and objective:**

PET/CT imaging is expensive, so searching the tumor imaging agent for SPECT/CT is necessary. ^99^Tc^m^-N(NOEt)_2_ [bis (N-ethoxy-N-ethyl dithiocarbamato) nitrido^99^Tc^m^ (Ⅴ)] can be uptaken by lung cancer cells and other cells alike. The aim of this study is to evaluate the distinctive value in lung tumor with ^99^Tc^m^-N(NOEt)_2_, the difference in its uptake kinetics in human embryonic lung diploid fibroblasts KMB17 and several kinds of lung cancer cells lines.

**Methods:**

Firstly, six different cell culture medium which contained YTMLC Gejiu human lung squamous carcinoma cell, SPC-A1 human lung adenocarcinoma cell, AGZY low metastatic human lung adenocarcinoma, 973 high metastatic human lung adenocarcinoma cell, GLC-82 Gejiu human lung adenocarcinoma cell, and KMB17 human embryonic lung diploid fibroblast, respectively with equal cell density of 1×10^6^/mL and the same volume were prepared; secondly, the same radioactive dose of ^99^Tc^m^-N(NOEt)_2_ was added into each sample and then 300 μL mixed sample was taken out respectively and cultured in 37 ℃ culture box; Finally, 5 min, 15 min, 30 min, 45 min, 60 min, 75 min, 90 min after cultivation, centrifuged each cultured sample and determined the intracellular radiocounts of each sample, calculated each cell sample's uptake rate of ^99^Tc^m^N(NOEt)_2_ at different time.

**Results:**

Statistical difference was found among six cell samples, and the uptake rate sequence from high to low is 973 and SPC-A1 > YTMLC > GLC-82 > AGZY > KMB17 respectively; furthermore, 30 min-45 min after culture, the uptake rate reached stability, and the 45 min uptake rate of each sample was higher than its 96.7% uptake peak.

**Conclusion:**

Based on the results above mentioned, it is supposed that there are discriminative clinical value when using ^99^Tc^m^-N(NOEt)_2_ as a tumor targeting imaging agent, and 30 min or so after injection may be the best imaging time in the early imaging stage.

^18^F-脱氧葡萄糖（fluorodeoxyglucose, FDG）PET/CT显像对良、恶性肿瘤的鉴别诊断和临床分期有独特和重要的价值^[1, 2]^，但其昂贵的仪器设备、药物和检查费用均限制了它的临床应用和推广^[3, 4]^；而SPECT/CT也具有较高的检测灵敏度、图像分辨率和准确定位，同时临床检查费用低，普及率高，易于推广和接受^[5, 6]^，因此研究^99^Tc^m^（锝）等标记的亲肿瘤显像剂是非常有必要的。

肺癌是我国发病率和死亡率最高的恶性肿瘤，^99^Tc^m^ -N(NOEt)_2_是一种非特异性亲肿瘤阳性显像剂，已被用于肺肿瘤等显像的研究^[7, 8]^，但肺炎性假瘤、肉芽肿、结节病、结核等良性病灶常导致假阳性，影响临床肺癌诊断的准确性，而这些良性病变内常含有较多成纤维细胞。为此，本研究对多种肺癌细胞及肺癌细胞与成纤维细胞间^99^Tc^m^-N(NOEt)_2_摄取动力学进行了比较研究，在细胞水平探讨其用于肺肿瘤显像的价值。

## 1 材料与方法

### 1.1 ^99^Tc^m^-N(NOEt)_2_的制备与质量控制

由^99^Mo-^99^Tc^m^发生器（中国原子能科学研究所原子高科股份有限公司）在（洗脱效率、淋洗间隔时间等）相似条件下新鲜淋洗的^99^Tc^m^O_4_^-^，加入N(NOEt)_2_药盒（北京师范大学师宏药物中心），按说明书制备^99^Tc^m^-N(NOEt)_2_，放化纯 > 90%方可使用，稀释至放射性浓度为3.7×10^7^ Bq/mL。

### 1.2 细胞培养

YTMLC个旧人肺鳞癌细胞、SPC-A1人肺腺癌细胞、AGZY低转移人肺腺癌细胞、973人高转移肺腺癌细胞及GLC-82个旧人肺腺癌细胞由本院肿瘤研究所提供；KMB17人胚肺二倍体成纤维细胞由中国医学科学院中国协和医科大学医学生物研究所（昆明）惠赠，所有细胞均由本院临床实验中心培养。培养液为含质量分数为15%小牛血清的RPMI-1640（含100 U/mL青霉素和100 μg/mL链霉素），培养于37 ℃、体积分数5%CO_2_培养箱中。将对数生长期的肿瘤细胞用质量分数为0.25%胰蛋白酶消化，吹打成单个悬浮细胞，计数并及时使用。细胞悬浮于无血清的RPMI-1640中，细胞浓度为1×10^6^/mL。实验前和实验中用台盼蓝排除法检测，细胞活力需 > 85%。

### 1.3 细胞摄取实验

塑料试管均先用含质量分数0.5%小牛血清的PBS浸泡1 h，然后用PBS洗涤3遍以减少非特异性吸附。将50 μL（1.85 MBq）^99^Tc^m^-N(NOEt)_2_分别加入8 mL含8×10^6^个细胞的KMB17人胚肺二倍体成纤维细胞及各种肺癌细胞培养瓶中，振荡混匀，迅速准确取样300 μL分别加入预处理好的塑料试管中，置于37 ℃恒温培养，于5 min、15 min、30 min、45 min、60 min、75 min、90 min分别取出，600 rpm离心3 min，弃上清，用FH-408自动定标仪（国营二六一厂）检测放射性。每个时间点设非特异性吸附管，即空白实验（方法同上，只是不含细胞）；总计数管3个（不需离心）。

### 1.4 数据处理

摄取实验结果用摄取百分率表示：特异性细胞净摄取放射性计数（沉淀放射性计数-空白放射性计数）/总放射性计数（300 μL细胞悬液放射性计数）。所有数据点取3个平行样本，每个样本重复测量3次。全部资料用Excel和SPSS录入和统计分析。正态分布资料用Mean±SD表示；采用重复测量资料的方差分析（repeated measures analysis of variance）分析不同细胞的摄取率情况。多个样本均数间的两两比较采用最小有意义差异*t*检验（least significant difference-*t* test, *LSD-t*）检验分析。以*P* < 0.05为有统计学差异。

## 2 结果

### 2.1 细胞系间摄取率差异比较

6种细胞（KMB17人胚肺二倍体成纤维细胞和5种肺癌细胞）^99^Tc^m^-N(NOEt)_2_摄取率的差异有统计学意义（*F*_药物_=1 584.926，*P* < 0.001），其中，除SPC-A1细胞与973细胞摄取均数间多重比较的差异无统计学意义外（*LSD-t*检验，*P*=0.838），其余两两之间的差异均有统计学意义（*P* < 0.001）。6种细胞的摄取率大小关系为：973与SPC-A1 > YTMLC > GLC-82 > AGZY > 成纤维。

### 2.2 各测量时间点摄取率差异比较

各次重复测量间变化趋势分析表明，7个测量时间的细胞摄取率差异有统计学意义（*Pillai's*轨迹检验*F*=5 016.575，*P* < 0.001）。除60 min与75 min、75 min与90 min之间的差异无统计学意义外（*LSD-t*检验，*P*≥0.343），其余两两之间的差异均有统计学意义（*P*≤0.006），即5 min、15 min、30 min、45 min、60 min的细胞摄取率逐渐升高，从60 min开始平缓，即60 min、75 min、90 min的细胞摄取率波动不大。

### 2.3 细胞种类与时间的交互作用

数据分析显示有统计学意义（*Pillai's*轨迹检验*F*_细胞×时间_=3.863，*P* < 0.001），细胞种类与时间之间存在交互作用（表 1）。

**1:** ^99^Tc^m^-N(NOEt)_2_在KMB17人胚肺二倍体成纤维细胞和5种肺癌细胞中的摄取率 The uptake rate of ^99^Tc^m^-N(NOEt)_2_ in KMB17 human embryonic lung diploid fibroblasts and five kinds of lung cancer cells

Time of measurement	Uptake rate of various cell (Mean±SD, %)					
	YTMLC	SPC-A1	AGZY	973	GLC-82	KMB17
5 min	8.415±0.110	9.157±0.166	8.353±0.335	9.031±0.100	9.000±0.146	8.100±0.016
15 min	12.873±0.267	14.932±0.104	10.995±0.376	14.022±0.157	13.272±0.635	10.200±0.018
30 min	16.930±0.080	16.965±0.182	13.745±0.250	16.329±0.426	15.000±0.048	11.287±0.273
45 min	17.139±0.062	17.337±0.181	14.484±0.434	17.614±0.173	15.664±0.372	12.484±0.021
60 min	17.624±0.007	17.522±0.024	14.307±0.179	17.912±0.364	16.143±0.063	13.400±0.091
75 min	17.681±0.091	17.512±0.031	14.493±0.730	18.048±0.083	16.116±0.045	13.541±0.052
90 min	17.681±0.114	17.724±0.098	14.627±0.099	18.105±0.043	16.162±0.041	13.509±0.040

## 3 讨论

^99^Tc^m^-N(NOEt)_2_是一种中性脂溶性心肌灌注显像剂，De Beco等^[9]^报道^99^Tc^m^-N(NOEt)_2_在肿瘤细胞也有摄取，在细胞内浓聚的机理尚不清楚。Johnson等^[10]^研究提示^99^Tc^m^-N(NOEt)_2_主要局限于细胞膜上或细胞膜内。体外研究^[11]^表明，肿癌细胞摄取^99^Tc^m^-N(NOEt)_2_可能是简单的弥散过程，不依赖温度，其能被肿瘤细胞摄取且摄取率明显高于^99^Tc^m^-MIBI [^99^Tc^m^-hexakis-2-methoxyisobutylisonitrile]，^99^锝^m^-六（2-甲氧基-2-异丁基异腈）]。其在肺肿瘤显像的研究^[7, 8]^显示诊断准确性达82.05%-85.00%，但与^99^Tc^m^-MIBI和^18^F-FDG相似也可被肺的炎性假瘤、肉芽肿、结节病、结核等肺良性病变摄取，造成假阳性^[7, 8, 12, 13]^，肺部呈阳性的良性病变组织中常存在大量的功能活跃的成纤维细胞、肌成纤维细胞和血管增生、管腔的扩张^[14]^。本研究中KMB17是一种人胚肺成纤维细胞，对KMB17成纤维细胞以及YTMLC个旧人肺鳞癌细胞、SPC-A1人肺腺癌细胞、AGZY低转移人肺腺癌细胞、973人高转移肺腺癌细胞及GLC-82个旧人肺腺癌细胞摄取^99^Tc^m^-N(NOEt)_2_的情况作对比研究，可以在一定程度上比较接近地反映肺炎性假瘤等肺良性病变和多种肺癌对^99^Tc^m^-N(NOEt)_2_摄取情况；同时，本研究中各种细胞的浓度均被调整为1×10^6^/mL，振荡混匀状态每个样本取量300 μL，相同体积的细胞悬液所含细胞数相同，而且各种培养液体积、蛋白含量、电解质浓度以及^99^Tc^m^-N(NOEt)_2_放射性浓度、放化纯及N(NOEt)_2_总含量也相同或比较相似，因此6种细胞的^99^Tc^m^-N(NOEt)_2_摄取动力学有可比性。

本研究结果显示：各种细胞30 min-45 min时摄取率逐渐趋于平稳，45 min摄取率均值约为摄取峰值均值的96.7%，提示临床早期摄取观察点应在45 min左右。考虑体内药物不断地被洗脱和代谢排出等因素的影响，早期显像在30 min是适合的。^99^Tc^m^-N(NOEt)_2_在KMB17成纤维细胞和5种肺癌细胞摄取率有统计学差异，大小关系为973与SPC-A1 > YTMLC > GLC-82 > AGZY > KMB17，KMB17在6种细胞中摄取率最低，因此，从本研究细胞摄取的层面显示：^99^Tc^m^-N(NOEt)_2_显像对肺肿瘤良、恶性的鉴别诊断是有应用价值的；当然，也注意到AGZY人肺腺癌细胞摄取峰值与KMB17成纤维细胞摄取峰值比值仅为1.082，这可能与本研究中的KMB17成纤维细胞处于对数生长期、功能较活跃有关；这也提示如果良性肺肿瘤中的成纤维细胞功能比较活跃，将可能导致诊断时出现假阳性或假阴性。临床上肺内异常活跃增生的病灶更容易发展为恶性病变，对这部分病变的检出在一定程度上阻断恶性转归，减少肺恶性肿瘤的发生。所以，^99^Tc^m^-N(NOEt)_2_显像用于肺肿瘤良、恶性的鉴别诊断具有一定的临床应用价值。

本研究主要从离体细胞对^99^Tc^m^-N(NOEt)_2_的摄取动力学方面阐释了肺癌细胞与成纤维细胞的摄取差异，而体内^99^Tc^m^-N(NOEt)_2_被肿瘤组织摄取和排出非常复杂。实验结果也显示细胞种类与时间有交互作用，细胞摄取率随时间延长而增加，不同时间细胞摄取增加的程度并不相同，提示各种细胞对^99^Tc^m^-N(NOEt)_2_的摄取除了与细胞本身的功能有关外，可能还与^99^Tc^m^-N(NOEt)_2_在肿瘤细胞简单弥散^[11]^程度、细胞膜Ca^2+^通道、P-糖蛋白表达等^[8]^影响摄取率的各种因素有关。因此对肿瘤的生长、代谢、血流灌注情况、瘤细胞的活力、增殖程度、P-gp肿瘤耐药基因或蛋白的表达^[8, 9]^以及良、恶性病变对显像剂的洗脱差异等多种因素影响，尚需进一步作临床和实验研究。
